# Interaction of the amyloid *β* peptide with sodium dodecyl sulfate as a membrane-mimicking detergent

**DOI:** 10.1007/s10867-016-9408-5

**Published:** 2016-03-16

**Authors:** Maryam Hashemi Shabestari, Nico J. Meeuwenoord, Dmitri. V. Filippov, Martina Huber

**Affiliations:** 1 Department of Physics, Huygens-Kamerlingh Onnes Laboratory, P.O. Box 9504, 2300 RA Leiden, The Netherlands; 2 Leiden Institute of Chemistry, Leiden University, NL-2300 RA Leiden, The Netherlands

**Keywords:** Amyloid membrane interaction, Amyloid-beta 1–40, Electron paramagnetic resonance (EPR), Alzheimer’s disease, Amyloid-beta SDS interaction

## Abstract

The amyloid *β* (A *β*) peptide is important in the context of Alzheimer’s disease, since it is one of the major components of the fibrils that constitute amyloid plaques. Agents that can influence fibril formation are important, and of those, membrane mimics are particularly relevant, because the hydrophobic part of A *β* suggests a possible membrane activity of the peptide. We employed spin-label EPR to investigate the aggregation process of A *β*1–40 in the presence of the sodium dodecyl sulfate (SDS) detergent as a membrane-mimicking agent. In this work, the effect of SDS on A *β* is studied using two positions of spin label, the N-terminus and position 26. By comparing the two label positions, the effect of local mobility of the spin label is eliminated, revealing A *β* aggregation in the SDS concentration regime below the critical micelle concentration (CMC). We demonstrate that, at low SDS concentrations, the N-terminus of A *β* participates in the solubilization, most likely by being located at the particle–water interface. At higher SDS concentrations, an SDS-solubilized state that is a precursor to the one A *β*/micelle state above the CMC of SDS prevails. We propose that A *β* is membrane active and that aggregates include SDS. This study reveals the unique potential of EPR in studying A *β* aggregation in the presence of detergent.

## Introduction

**Fig. 1:**
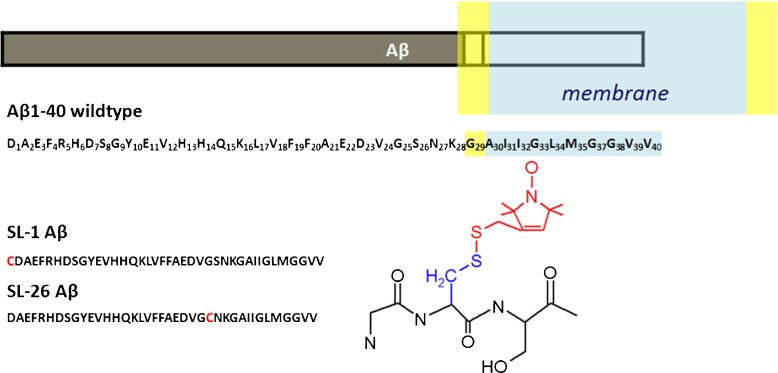
Overview of A *β*1-40 sequence and constructs. *Top*: Schematic of A *β*1–40 relative to membrane location of the amyloid precursor protein (APP) [3, 5–9], membrane dimensions not to scale. *Light blue*: hydrophobic part of the membrane, *yellow*: lipid headgroup region. *Middle*: A *β*1–40 sequence, *bottom left*: constructs used. *Red*: cysteine used to link MTSL spin label. *Bottom right*: MTSL-linked to a schematic protein backbone

The aggregation of amyloid *β* (A *β*) peptide to fibrils and plaques is the chief indicator of Alzheimer’s disease [1–6]. The peptide is derived from misprocessing of the amyloid precursor protein (APP) and comprises a part of the presumed transmembrane section of APP [3, 5–9], shown schematically in Fig. 1. The two major amyloid *β* fragments are the peptides comprising the 40 amino acids shown in Fig. 1, wildtype (wt) sequence, which is referred to as A *β*1–40, and A *β*1–42. The A *β*1-42 has an additional isoleucine I and an alanine A extending the C-terminus of A *β*1–40. In solution, the A *β* peptides are disordered and especially at high concentration their tendency to aggregate into fibrils is high [10].

In the fibrils, A *β* adopts a parallel, *β*-sheet structure [3, 11]. The potent pathologic effects of A *β* oligomers provide a compelling reason for elucidating the mechanism(s) leading to the transformation of monomeric A *β* into toxic oligomers and ultimately larger aggregates [5, 8, 12–16]. Furthermore, agents that can influence aggregation are important, as recently reviewed in Wärmländer et al. [17]. Membrane mimics are particularly relevant because membrane activity is one of the mechanisms by which A *β* could damage cells. The hydrophobic part of A *β*, indicated in Fig. 1, suggests a possible membrane activity of the peptide.

The sodium dodecyl sulfate (SDS) detergent is a commonly used membrane mimic in A *β* studies [18–23] because it can be solubilized with A *β* at any desired concentration, whereas lipids, which constitute real membranes, have to be added as solutions of preformed membrane preparations, such as vesicles. Especially low concentrations of the lipid and low lipid–peptide ratios are difficult to obtain, since lipids cannot easily be added to A *β*, unless co-solvents are used. Ternary mixtures of A *β* with co-solvents and lipids have complex membrane properties and are therefore avoided. In the present study, we use SDS because we are interested in the A *β* aggregation over the entire concentration regime of the lipid/detergent, particularly low concentrations, and such studies cannot be performed with lipids. Also, using SDS enables us to directly compare with solution NMR, a method that cannot be applied to lipid vesicles. The price to be paid is that detergents form micelles [24–29], rather than the lipid bilayer membranes that constitute vesicles. Therefore, whenever we refer in the following to SDS as a membrane-mimicking agent it is meant in the limited sense described above.

The aggregation of A *β* under the influence of SDS concerns two concentration regimes [21]. At low concentrations of SDS or low SDS to peptide ratios (D/P), evidence for aggregates was found. These aggregates appeared to have a *β*-sheet component [16, 20, 21], suggesting aggregates that possess the secondary structure element of A *β* in the fibrils. Two different *β*-sheet containing species were proposed, one present at D/P ratios lower than 11, the other above D/P 11. In this SDS concentration regime, no solution NMR signals were detected [21], therefore molecular-detail information about these species is lacking.

Recent small angle X-ray scattering (SAXS) data show that the *β*-sheet oligomers at 0.9 mM SDS, i.e., below D/P 11, fit a two-cylinder shape with a cylinder radius of 2.7 nm and are distinct from fibrils [23, 30], and Sambasivam et al. propose from FRET distances between residues 1 and 10 that the N-terminus is an *α*-helix or a *β*-turn rather than an extended *β*-sheet [31]. Monomeric A *β*, which can be detected by NMR under these conditions, is in fast exchange with the aggregates, which, by themselves, are NMR-invisible [23]. At higher SDS concentrations, i.e., the concentration range at and above the critical micelle concentration (CMC) of SDS in water [24–27, 29] solution NMR signals reappear and A *β* is found to have an *α*-helical conformation. A detailed study using solution NMR [21, 32] revealed that A *β* is monomeric and embedded in an SDS micelle, a model that is supported also by small-angle X-ray and neutron scattering, FTIR, and CD spectroscopy [16, 21, 32–38].

Methods that can obtain molecular detail over the entire SDS concentration regime are sought to unravel how A *β* interacts with lipid mimics and how it is arranged under the various D/P-regimes. The tool we use is spin-label EPR, which has been used before in A *β* research [39–41]. For example, it was shown that signatures of the oligomeric A *β* peptide can be detected by the spin-label EPR methodology [42]. Measurements on tethered A *β*1-40 were performed to determine monomer properties of this peptide [43]. Isolated A *β*1-42 oligomers were characterized, showing that A *β*-peptides arrange in antiparallel *β*-sheets and have a mobile N-terminus and a less mobile middle section [44]. Also, the interaction of A *β*-oligomers with other proteins [45] and fibril structure [46] was tested by EPR. Here, we study A *β* in the entire concentration regime of SDS, from low SDS concentrations (D/P = 2.7) to conditions where SDS micelles should be present (D/P = 131), at peptide concentrations that promote fast and irreversible aggregation in the absence of SDS. The constructs we investigate are based on the A *β*1-40 sequence, abbreviated in the following as A *β*40. The nitroxide spin label is attached to cysteines introduced into the A *β*40 sequence, one at the N-terminus (SL1-A *β*) and, in the second construct, in the middle of the sequence (SL26-A *β*), see Fig. 1. To avoid line broadening by spin–spin interactions, we use diamagnetic dilution [3, 42, 47]. Diamagnetic dilution refers to diluting the spin-labeled A *β* peptide (SL-A *β*) with unlabeled A *β* peptide (wild-type A *β*). These A *β*-mixtures are incubated with SDS at various concentrations. The SDS concentrations were chosen to overlap with the detergent/peptide (D/P) ratios employed by Wahlström et al. [21].

We show that by EPR we obtain information on the state of A *β* in situ, and over the entire SDS concentration regime. In particular, we address the NMR-silent regime at intermediate SDS concentrations and provide structural features of the two *β*-sheet forms at these SDS concentrations. At SDS concentrations above the CMC, the monomeric A *β*40 bound to the micelle state, also found in previous studies [21, 23], is recovered. In the NMR-blind regime at lower SDS concentrations we show that peptide-detergent aggregates are formed, in which the shape and location of the A *β* depends strongly on the detergent–peptide (D/P) ratio. Apparently, the N-terminus is involved in these aggregates. We also propose detergent-like action of A *β* at low SDS concentrations.

## Materials and methods

The A *β*40 peptide as well as two cysteine-A *β*40 variants: [cys26] –A *β*40 and [cys1] –A *β*40, differing in the position of the spin label, were purchased from AnaSpec (purity > 95%), the solvent DMSO was purchased from Biosolve (purity 99.8%), the spin probe MTSSL [1-Oxyl-2,2,5,5-Tetramethyl- Δ-Pyrroline-3-Methyl] Methanethiosulfonate was purchased from Toronto Research Chemicals Inc. (Brisbane Rd., North York, Ontario, Canada, M3J 2J8). Spin labeling was performed and the purified spin-labeled A *β* was analyzed by liquid chromatography and liquid chromatography/mass spectrometry as described previously [42]. The spin-labeled construct thus obtained is referred to as SL1-A *β* or SL26-A *β*. The peptide was lyophilized and stored in the freezer (–20°C) until used.

### Sample preparation protocol

Two cysteine variants of the A *β* peptide, SL1-A *β* and SL26-A *β*, varying in the position of the spin label were used. From each A *β* peptide variant, six different A *β* sample conditions, differing in SDS concentrations (1.5, 3, 4, 7, 36, and 72 mM) were prepared and compared to a sample to which no SDS was added. The total peptide concentration was kept constant at 0.55 mM. The peptide was a mixture of wild-type A *β* and SL-A *β*, which contained 14% SL-A *β*, resulting in diamagnetically diluted samples as reported before [42]. In contrast to the previous protocol [42], we prepared the A *β* samples using a procedure which involves predissolution of the peptide in dilute base solution [21, 48, 49]. This procedure was designed to avoid peptide aggregation in the starting solution.

Accordingly, the A *β* peptides were predissolved in NaOH solution (10 mM, pH 11) with sonication for 1 min in an ice bath at twice the desired final concentration, i.e., at 1.1 mM total A *β* concentration. The desired amount of SDS was dissolved in potassium phosphate buffer (20 mM, pH 7.4). The basic solution of A *β* peptides (1.1 mM) was combined with the potassium phosphate buffer solution (20 mM, pH 7.4) to reach the final desired peptide concentration and the proper detergent to peptide (D/P) molar ratio for each sample. In the remainder of the text, we use the detergent-to-peptide (D/P) ratio to refer to each sample condition, i.e., D/P = 0, 2.7, 5.4, 7.3, 12.7, 65.4, and 130.9, which refers to [SDS] = 0, 1.5, 3, 4, 7, 36, and 72 mM, respectively (see Table 1). This step was followed by another 1-min sonication in an ice bath. The final pH was adjusted to pH 7.4. The entire sample preparation was performed on ice and took a few minutes. All samples were prepared and measured at least twice. To make sure that the sample state did not change in the accumulation period, the EPR signal amplitude was occasionally checked after the accumulation. Measurements on aged samples, e.g., after 10 days or 2 weeks, did not reveal any change in the EPR spectra.

**Table 1:** Correspondence of SDS content of samples. Ratio of SDS detergent to A *β* peptide (D/P) and corresponding absolute SDS concentrations

D/P ratio	SDS [mM]
0	0
2.7	1.5
5.4	3
7.3	4
12.7	7
65.4	36
130.9	72

### EPR experiments

The X-band continuous wave (cw) EPR measurements have been performed at room temperature (20°C) using an ELEXSYS E680 spectrometer (Bruker, Rheinstetten, Germany) equipped with a rectangular cavity. Samples of 10–15 *μ*l peptide solution were drawn into Blaubrand 50- *μ*l capillaries. Often, a white precipitate was observed. In cases where a white precipitate was observed, the sample height was carefully adjusted in order to be sensitive to that part of the solution. Measurements were performed using the following parameters: 6.31 mW of microwave power, a modulation amplitude of 1.4 G, and a modulation frequency of 100 kHz. The large modulation amplitude helps to obtain a better signal-to-noise ratio for broad lines. The accumulation time for the spectra was 40 min per spectrum.

### The amount of spin label in different samples

For a quantitative comparison of samples, we need to investigate the actual amount of spin label in each sample. This amount was determined by double integration of the first-derivative EPR spectrum, with the SL-A *β* stock solution as a reference. The amount of spin label for the samples with different concentrations of SDS was at least 86% compared to the stock solution. The uncertainties of this method, determined by multiple independent analyses of the same data, are around 20% due to difficulties with the baseline correction of the spectra. Within this error margin, the amount of spin-labeled peptides in all samples is identical.

### Simulations of EPR spectra

MATLAB (version 7.11.0.584, Natick, MA, USA) and the EasySpin package [50] were used for the simulation of EPR spectra. For all simulations, the following tensor values were used: g = [2.00906, 2.00687, 2.00300] [42, 51] and A_xx_ = A_yy_= 12 and 13 MHz in DMSO and buffer, respectively. For the fast and medium components, different A_zz_ values were used than for the slow component, as discussed before [42]. For each fraction, over-modulation effects were taken into account in EasySpin. Usually, a superposition of 1–3 components was required to simulate the spectra. In all cases, isotropic rotation of the spin label was sufficient to reproduce the line shape observed.

We interpreted *τ*_r_ with the Stokes–Einstein equation, which implies a spherical approximation for the volume [42]:

1 $$ \tau_{r} =\frac{4\pi \eta \alpha^{3}}{3kT}=\frac{\eta }{kT}V_{EPR}.  $$

The Boltzmann constant, k, and solvent viscosity, *η*, at a specified temperature, T, are required to obtain the hydrodynamic radius, *α*. According to (1), the volume, V_EPR_, of the particle is linearly correlated with the *τ*_r_ of the spin-labeled peptide. The volumes derived are referred to as V_EPR_ in the text. The volume V_EPR_ derived from *τ*_r_ is strongly affected by the mobility of the nitroxide group of the spin label and the rotation of the spin label around the linker bond can make this correlation time significantly smaller than that of the aggregate.

## Results

**Fig. 2:**
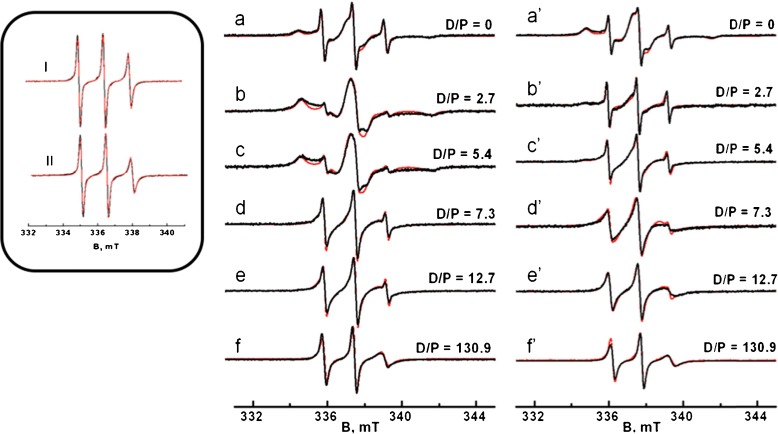
Room-temperature EPR spectra of SL1-A *β* and SL26-A *β* in PBS for samples with different SDS detergent to peptide (D/P) ratios. **a**–**f** Spectra for SL1-A *β* samples. From **a** to **f**, the D/P ratio increases. **a**’ to **f**’: Spectra for SL26-A *β* samples organized as in the left part of the figure. *Black line*: experiment, *red line*: simulation. The *inset* shows the spectra in DMSO where A *β* is monomeric. I: SL1-A *β* (rotation-correlation time *τ*_r_ 0.19 ns), II: SL26-A *β* (*τ*_r_ 0.27 ns)

The spectra of both SL-A *β* variants in DMSO, in which the A *β* peptide is in the monomeric form [52–54], have three narrow lines (Fig. 2, inset). At low field, the first two lines of both SL-A *β* variants in DMSO have similar intensities, whereas the intensity of the third line at high field is larger for the sample of SL1-A *β* compared to that of SL26-A *β*.

Under aggregation conditions [42], i.e., in buffer and in the absence of SDS (D/P = 0), the lines of both SL-A *β* variants are broadened and additional lines are observed as reported before [42] (Fig. 2a and 2a’). Under these conditions, fibrils were detected by transmission electron microscopy [42]. In the presence of SDS, particularly at low concentrations of SDS (D/P = 2.7, 5.4), the spectra of SL1-A *β* differ from those of SL26-A *β* (Fig. 2a–c and 2a’–c’), whereas at higher concentrations (above 7 mM, D/P = 12.7), both SL-A *β* variants have identical spectra (Fig. 2f and 2f’). At D/P ratios of 7.3 and 12.7, the spectrum of SL1-A *β* has narrower lines compared to those of SL26-A *β*.

By means of simulation, we quantify the spectral changes. The spectra of both SL-A *β* variants in DMSO are simulated by a single component with a *τ*_r_ value of 0.19 ns for SL1-A *β* and 0.27 ns for SL26-A *β*. So even though the SL1-A *β* sequence is longer by one residue than SL26-A *β*, SL1-A *β* has a shorter correlation time. We attribute the difference in the *τ*_r_ values to a slightly lower local mobility of the spin label at position 26 compared to that at position 1. The spectra of both SL-A *β* variants in PBS and in the absence of SDS detergent are simulated using three components which, in the remainder of the text, we refer to as fast, medium, and slow. Each component is characterized by its *τ*_r_ value, and the amount by which this component contributes to the spectrum (Tables 2 and 3). As discussed previously [42], including additional components in the fit does not significantly improve the agreement with the experimental data. Therefore the spectra are interpreted as containing three components or less. The *τ*_r_ values of the components agree well with those found previously [42]. In the present study, a larger amount of monomeric A *β* (10 vs. 5%) was found compared to [42], which we ascribe to the different preparation protocol [21, 48, 49], a protocol that was designed to increase the amount of monomeric A *β*.

**Table 2:** EPR parameters obtained from the simulation of cw EPR spectra of the SL1-A *β* samples

	Fast				Medium				Slow			
D/P	*τ*_r_^a^	A_zz_	lw	%^b^	*τ*_r_	A_zz_	lw	%	*τ*_r_	A_zz_	lw	%^c^
	(ns)	(MHz)	(MHz)		(ns)	(MHz)	(MHz)		(ns)	(MHz)	(MHz)	
0	0.19	110	0.14	10	2.55 ± 0.35	110	0.32	51	> 50	95	0.50	39
2.7	0.43	110	0.14	2.5	4.80 ± 0.40	110	0.32	64	> 50	95	0.50	33.5
5.4	0.43	110	0.14	2.5	4.65 ± 0.55	110	0.32	75	> 50	95	0.50	22.5
7.3	0.19	110	0.14	10	1.76 ± 0.16	110	0.14	90	–	–	–	–
12.7	0.19	110	0.14	7	1.55 ± 0.08	110	0.14	92	–	–	–	–
65.4	–	–	–	–	0.93 ± 0.03	110	0.06	100	–	–	–	–
130.9	–	–	–	–	0.93 ± 0.03	110	0.06	100	–	–	–	–

Given are: *τ*_r_, rotation-correlation time, A_zz_, the hyperfine splitting along the *z*-direction, lw, the component line width of the simulation and % stands for the contribution of the component to the total spectrum

^a^ Errors: ± 0.02 ns

^b^ Errors: ± 1%

^c^ Errors: ± 4.00%

**Table 3:** EPR parameters obtained from the simulation of cw EPR spectra of the SL26-A *β* samples

	Fast				Medium				Slow			
D/P	*τ*_r_^a^	A_zz_	lw	%^b^	*τ*_r_	A_zz_	lw	% ^*b*^	*τ*_r_	A_zz_	lw	%^c^
	(ns)	(MHz)	(MHz)		(ns)	(MHz)	(MHz)		(ns)	(MHz)	(MHz)	
0	0.27	110	0.14	6	3.6 ±0.10	110	0.32	52	> 50	95	0.50	42
2.7	0.26	110	0.14	24	2.1 ±0.10	110	0.32	36	> 50	95	0.50	40
5.4	0.26	110	0.14	13	2.1 ±0.10	110	0.32	74	> 50	95	0.50	13
7.3	0.26	110	0.14	4	2.1 ±0.10	110	0.14	96	–	–	–	–
12.7	0.27	110	0.14	7	1.4 ±0.10	110	0.14	93	–	–	–	–
65.4	–	–	–	–	0.93 ±0.03	110	0.06	100	–	–	–	–
130.9	–	–	–	–	0.93 ±0.03	110	0.06	100	–	–	–	–

Given are: *τ*_r_, rotation-correlation time, A_zz_, the hyperfine splitting along the *z*-direction, lw, the component line width of the simulation and % stands for the contribution of the component to the total spectrum

^a^ Errors: ± 0.02 ns

^b^ Errors: ± 1%

^c^ Errors: ± 4.00%

To illustrate the sensitivity of the EPR line shape to the rotation correlation time, it is instructive to compare the spectra I, II, f and f’ in Fig. 2. These spectra concern a single mobility component each, and the respective correlation times are 0.19 ns (spectrum I), 0.27 ns (spectrum II) and 0.93 ns (spectra f and f’). Comparing I and II, a broadening of the high-field line is visible from the decrease in line intensity, showing that differences of 80 ps in rotation correlation times already cause differences in line shapes that can discerned by the naked eye. Obviously, also the larger differences in rotation correlation times of I and II with respect to f and f’ is easy to distinguish by the broadening of the high-field line.

In the following, we first explain the interpretation of the rotation correlation times, then describe development of the amount of the three mobility fractions, and next the corresponding *τ*_r_ *values*_._

### Interpretation of rotation correlation times

According to the Stokes–Einstein equation, Eq. 1, the volume of the particle has a linear dependence on *τ*_r_(see Section 2), therefore, from *τ*_r_ we can determine the EPR derived volume of the aggregates, V_EPR_, (see Section 2). For the volume of the slow component (*τ*_r_ > 50 ns), only a lower limit of 48,000 Å ^3^could be given because this component is immobile on the time scale of the EPR experiment. For the fast-rotating fraction of the sample with D/P = 0, a *τ*_r_ of 0.19 ns and 0.27 ns for SL1-A *β* and SL26-A *β* is obtained, respectively. Using the viscosity of water of *η* = 1.002*10 ^−3^ (N ⋅*s*⋅*m* ^−2^) at 20°C [55], a volume of 180 Å ^3^ results, which is close to the volume of 126 Å ^3^ obtained from the *τ*_r_ of A *β* in DMSO (*η* = 1.996*10 ^−3^ N ⋅*s*⋅*m* ^−2^[55], *τ*_r_ = 0.26 ns) in which the peptide is in the monomeric form. Following Sepkhanova et al., the fast component is assigned to the monomeric peptide [42]. As described before, the *τ*_r_ values contain a contribution of the local mobility of the spin label that is due to the rotation of nitroxide-containing ring about the single bonds by which the nitroxide is attached to the peptide backbone (see Fig. 1). This motion is fast compared to the rotation of the aggregates, and therefore dominates the *τ*_r_ values. Therefore, *τ*_r_ values are sensitive reporters of the local environment of the spin label, but not of the size of the aggregate.

### Effect of SDS on the amount of different components

**Fig. 3:**
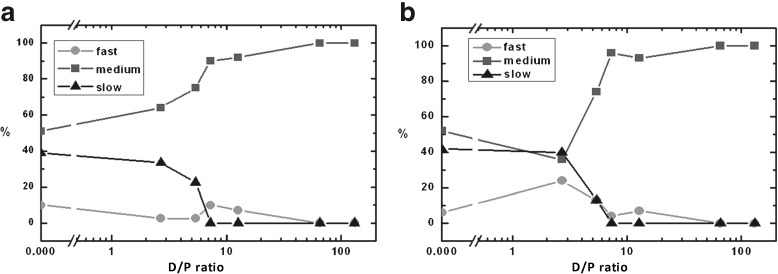
Amount of the spectral components as a function of the SDS concentration, expressed as the D/P ratio. **a** SL1-A *β*. **b**SL26-A *β*

Figure 3 shows the development of the amount by which each mobility component contributes to the spectra as a function of the SDS concentration. In the absence of SDS (D/P = 0), the spectra of both SL-A *β* variants are composed of almost equal amounts of the slow and the medium component and a small amount (about 10%) of the fast component. At low concentrations of SDS (between D/P = 0 and 5.4), the development of the amount of the fast and the medium components of SL26-A *β* is different from that of SL1-A *β*. For SL1-A *β* the amount of fast component decreases and the amount of the medium component increases, whereas SL26-A *β* shows the opposite trend (Fig. 3). In the same concentration region (between D/P = 0 and 5.4), the amount of the slow component decreases in both SL-A *β* variants. Above a D/P ratio of 5.4, the slow component has disappeared, leaving only the fast and medium components. At higher concentrations of SDS (above 7 mM SDS, i.e., D/P = 12.7), which is close to the critical micelle concentration (CMC) of neat SDS in water [24–29], only one component of medium mobility is left, which has the same parameters for both SL-A *β* variants.

### Effect of SDS on the rotation correlation time

The *τ*_r_-values of the fast component of the EPR spectra of SL1-A *β* and SL26-A *β* in buffer are identical to those of the respective SL-A *β* variants in DMSO, showing that the fast fraction should be monomeric A *β*. In the presence of SDS, up to D/P = 5.4 the *τ*_r_-values of the fast and the medium component of SL1-A *β* are larger than those of SL26-A *β*. For SL1-A *β*, at D/P <7.3, the *τ*_r_-values of both fast and medium components slightly increase with increasing SDS concentration, whereas those for SL26-A *β* remain constant over that range (D/P <7.3). At higher values of D/P (above D/P = 12.7; i.e., 7 mM SDS), no fast component is detected in the spectra of both SL-A *β* variants. The *τ*_r_-values of the only observed component in both SL-A *β* variants are identical. This *τ*_r_ is longer than the *τ*_r_ of both SL-A *β* variants in DMSO, in which the A *β* peptide is in the monomeric form.

### Is the species observed at high SDS concentrations monomeric?

**Fig. 4:**
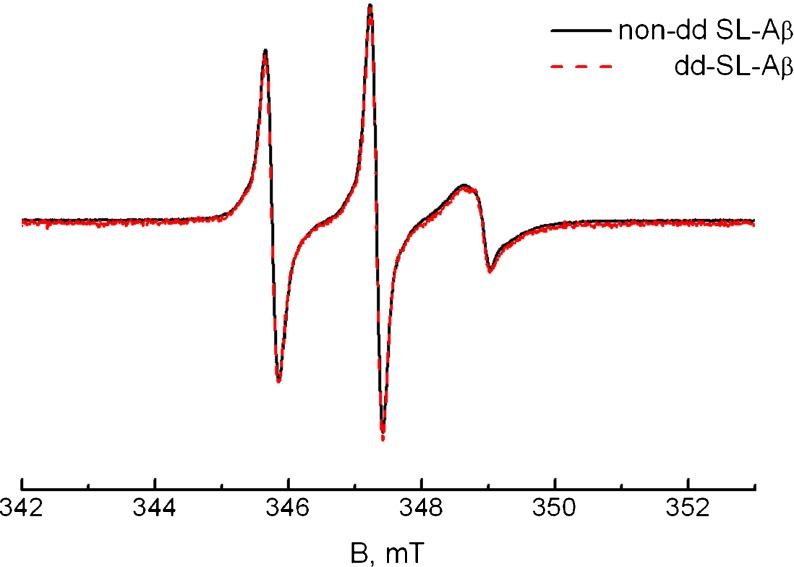
Spectra demonstrating the absence of spin–spin interaction in the high-SDS form of A *β*. At a D/P ratio of 130.9, pure SL1-A *β* (*black line*) has the same spectrum as diamagnetically diluted A *β* (*red, dashed line*: dd-SL1-A *β*)

To test for spin–spin interaction, we measured a pure SL-A *β* sample at a D/P ratio of 130.9. The result is shown in Fig. 4. There is no difference between the diamagnetically diluted and the non-diluted sample, showing that there is no spin–spin interaction between the A *β* peptides in that state.

## Discussion

We have investigated the properties of A *β* in the presence of different concentrations of SDS. We propose that the state of A *β* changes in a continuous fashion as a function of SDS concentration and that there are different types of aggregates at SDS concentrations below the CMC.

### The state of A *β* in the absence of SDS

In the absence of SDS, the EPR spectra consist of three components and match closely those described in our earlier study [42]. As described before [42], at these high concentrations of A *β*, the peptide aggregates fast. In the time of several minutes that it takes to prepare the sample, the peptide is already fully aggregated, and the EPR spectra do not change with time. According to transmission electron microscopy (TEM) on wt-A *β*40 and different mixtures of A *β*40 with SL-A *β*, which we had published earlier [42], aggregates and fibrils are present, and the solutions were Congo red active, confirming the presence of fibrils. We also proved that the three-component EPR spectra such as shown in Fig. 2a are correlated with fibrils [42]. Analogous to the previous study [42], we refer to the three components in the EPR spectra as the fast, medium, and slow components. We attribute the fast component to monomeric A *β* and the two others to aggregated forms of A *β*. Since both in buffer and in DMSO, monomeric SL1-A *β* has a smaller *τ*_r_than SL26-A *β*, we conclude that the central region of A *β* is less flexible than the N-terminus.

The ratio of the fractions shows that the largest portion of the sample is aggregated, as maximally 12% of the fast, monomeric fraction is found. This observation is in good agreement with previous reports on the concentration dependence of the A *β* aggregation [10, 21]. In A *β* preparations made specifically to keep A *β* monomeric by tethering, no EPR signals equivalent to our slow fraction are detected (Gu et al. [43]), emphasizing that the slow fraction is aggregated A *β*.

### The high-SDS state: A *β* at SDS concentrations above the CMC

At high concentrations of SDS, A *β* occurs as a single species, referred to as the high-SDS species. Since these SDS concentrations are well above the CMC of SDS in water [24–29], it stands to reason that SDS is in the micellar form also in the solutions investigated here. The high-SDS-A *β* species represents at least 80% of the total peptide in the sample (see Section 2) and is the only species we observe. Under these conditions, A *β* is monomeric, as proven by the absence of spin–spin interaction in spectra of pure, i.e., non-diamagnetically diluted, SL-A *β* (Fig. 4). The *τ*_r_ of this species is longer than that of the monomeric (fast) fraction of A *β* in the absence of SDS, showing that the spin-label of A *β* is interacting with the micelle. Even though longer than the *τ*_r_of the monomeric species, the *τ*_r_ never becomes as long as that expected for rotational diffusion of the SDS micelle [24, 56] or rotational diffusion times determined from NMR results of A *β* bound to micelles [21, 36, 38], showing that the spin label has local degrees of freedom. These local degrees of freedom derive from rotations around the single bonds linking the nitroxide to the peptide backbone (see Fig. 1). Identical *τ*_r_ values for the N-terminus and the central position of A *β* indicate that the N-terminus and the central part of A *β* have similar local interactions with the micelle. The idea of monomeric A *β* bound to a micelle is fully consistent with the results of other techniques [18, 21, 23, 32, 33, 35, 36, 38].

Our experiments, which show a homogeneous, non-interacting species of A *β* at these high SDS concentrations, cast new light on previous NMR-titration data, which, at similar D/P ratios, revealed that A *β* is heterogeneous, and only a fraction of about 20% of the A *β* was visible to NMR, i.e., monomeric [21]. The most likely explanation is that titration is less effective in breaking up aggregates than incubating A *β* directly with the final, high concentration of SDS, as done in the present study. A second factor could be that the absolute concentrations of NMR and EPR are not the same: The peptide concentration in the Section 2.2 is higher than in NMR, and consequently, the CMC of SDS is reached at lower D/P ratios than in the NMR experiments. This could help to favorably influence the equilibrium between A *β*-A *β* and A *β*-SDS interaction [18] and result in a larger fraction of monomeric A *β* bound to the micelle in the EPR compared to the NMR experiment. Further information from SAXS data suggests that A *β* is bound to the micelle-headgroup region [23].

### Development of aggregate species at SDS concentrations below the CMC

**Fig. 5:**
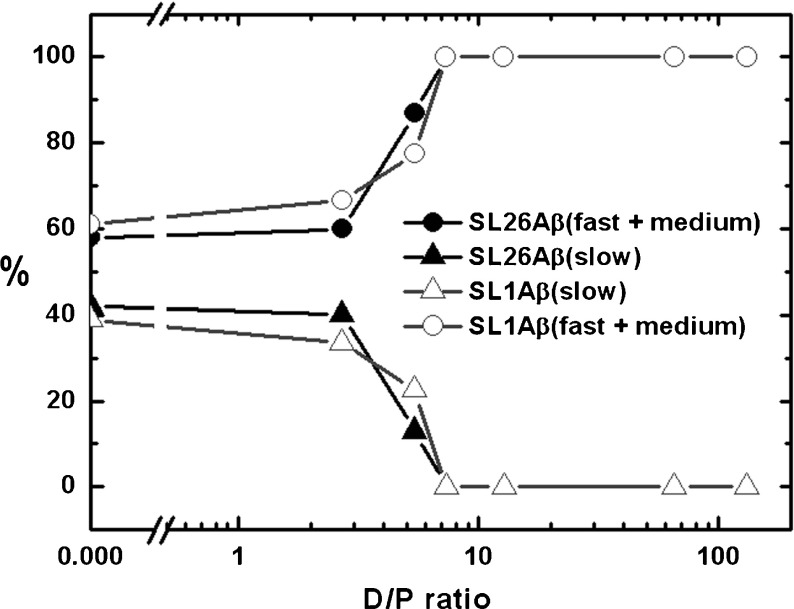
Amount of the spectral components as a function of the D/P ratios. The lines joining the points represent the amount of the slow (*filled triangles*: SL26-A *β*, *non-filled triangles*: SL1-A *β*) and combined fast and medium (*filled circles*: SL26-A *β*, *non-filled circles*: SL1-A *β*) components of the SL1-A *β* and SL26-A *β* variants, similar to Fig. 3. In both SL-A *β* variants, the amount of slow component decreases, whereas the amount of the more mobile components (fast plus medium) increases

In contrast to the interpretation of the high-SDS-species, much less is known so far about the state of the peptide at intermediate concentrations of SDS. To determine which aggregates are present at different SDS concentrations, we need to reduce the influence of the spin-label mobility on the data. Adding the amounts by which the fast and medium fractions contribute to the spectra at each SDS concentration such a measure is obtained, as shown in the plot in Fig. 5.

At each SDS concentration, the amount of the faster (fast + medium) and the slow fraction are identical for SL26-A *β* and SL1-A *β* within the experimental uncertainty, whereas the three fractions taken individually (see Fig. 3a vs. b) differ significantly. Evidently, the combined fractions in Fig. 5 reflect the state of the sample, rather than differences in local spin-label mobility. Between D/P ratios of two and ten, a steep increase in the faster fractions (fast + medium in Fig. 5) is accompanied by a decrease in the amount of the slow fraction, showing that the aggregation state is strongly dependent on the SDS concentration in that SDS regime. Also, Abelein et al. [23] find the largest changes of CD, pyrene, and fluorescence data in the narrow SDS concentration regime from 1 to 2 mM SDS. This is close to the absolute SDS concentrations at which we observe the most pronounced changes in the EPR spectra.

At the start of the region of the steepest change in the present study, i.e., at a D/P of 2.7, the combined fractions (Fig. 5) are similar to those in the pure A *β* state; however, locally, the A *β* seems to take on a different conformation than in the absence of SDS (Fig. 3a and b). The N-terminus, at a D/P of 2.7, is less mobile than in the absence of SDS, as evidenced by the larger *τ*_r_-values (Table 2) of the fast and medium component and the smaller amount of the fast component of SL1-A *β*. At low SDS-to-peptide ratios, aggregates should be dominated by A *β*–A *β* interactions and these apparently restrict the mobility of the N-terminus. Support for involvement of the N-terminus comes from Sambasivam et al. [31], who propose an *α*-helix or a *β*-turn for the N-terminus of A *β*, rather than an extended conformation such as a *β*-sheet. The central part of A *β* shows the opposite behavior. It is more mobile than in the absence of SDS and also more mobile than the N-terminus at this SDS concentration.

As described in Section 1, at SDS concentrations below the CMC, Wahlström et al. [21] find two *β*-sheet-type oligomeric structures, with a transition point around a D/P ratio of 11, i.e., an SDS concentration of 1.6 mM or D/P of 3 in our study. Our results suggest that in the first species, observed at lower SDS concentrations [21], the N-terminus is trapped in the aggregate. Since most [57–60], but not all [61] A *β* fibril models place the N-terminus outside the fibril core, this suggests a non-fibril-like *β*-sheet oligomer. Also, TEM and SAXS data argue against a fibril-like shape for these aggregates [23]. The results of NMR show that at these SDS concentrations the monomers are in fast exchange with the aggregates, which, by themselves, are NMR-invisible. On the EPR timescale, the fast mobility fraction, attributed to monomers, is in slow exchange with the aggregates, in agreement with faster time-scale of EPR compared to NMR. The structural features of A *β* in the different aggregation states are summarized in the next section.

### Indications for conformation of A *β* in the SDS oligomers

**Fig. 6:**
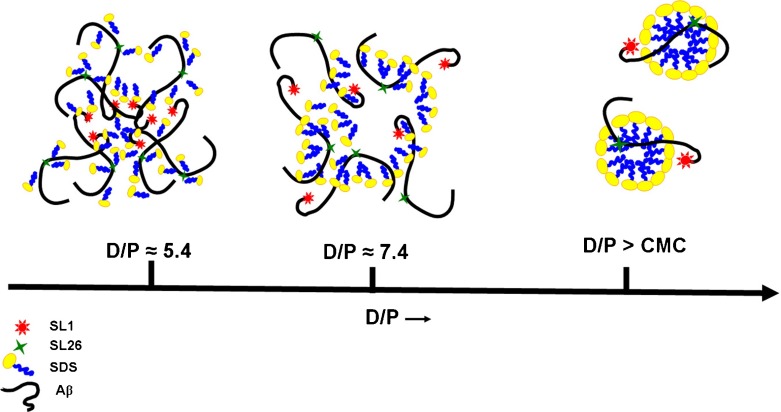
Illustration of the A *β* aggregation at different D/P ratios. On the *left side*, the A *β* aggregate is shown at a D/P of about 5.4, in which the hydrophilic N-terminus becomes immobilized at the aggregate–buffer interface. This helps to solubilize the aggregate. In the middle, the A *β* aggregate is shown at D/P ratios of about 7.3, where there are sufficient SDS molecules to replace (some of) the A *β* N-termini at the water–aggregate interface. On the *right*, the A *β* peptide is shown at D/P ratios above the CMC of SDS. Two possible models for A *β* interaction with a micelle are shown, in which both spin labels would have similar rotation correlation times

To illustrate our emerging view of A *β* aggregate development in the presence of SDS, we sketch structural features of A *β*-SDS interaction derived from EPR in Fig. 6. At lower D/P ratios, left hand sketch in Fig. 6 (for details, see figure caption), the N-terminus is buried in the aggregate. Since it is hydrophilic [62], and in most of the models [57–60] is not involved in the *β*-sheet area of the A *β*-fibrils, we propose that the most likely location of the N-terminus is at the water/aggregate interface, which helps to solubilize the aggregate. The sketch in Fig. 6 shows a possible way how the central part of A *β* could be more mobile under these conditions: The hydrophobic tails of SDS could bind to the hydrophobic aggregation domains of A *β* (residues 25-35) [63], while the hydrophilic head-groups of SDS enable solubilization towards the aqueous surroundings, thereby freeing the central region of A *β* from the aggregate. At intermediate D/P ratios, middle sketch in Fig. 6, the N-termini of A *β* are liberated from the aggregate, presumably because in that concentration regime there are sufficient SDS molecules to provide a hydrophilic cover for the aggregate. The central part of A *β*, however, becomes trapped in the centre of the A *β*-SDS aggregate. The right-side sketch in Fig. 6 illustrates the micelle-bound form of A *β*, the high-SDS species. Similar rotation-correlation times at the N-terminus and the central part of A *β* could derive from the N-terminus binding at the micelle-headgroup region of the micelle, while the central part is located in the middle of the micelle, where the ends of the tails of SDS could provide wriggling space.

According to Jarvet et al. [32], neither the N-terminus nor the central region of the peptide are part of the helical domain. Therefore, the partial immobilization we observe cannot be attributed to intramolecular interactions deriving from helix formation, but rather to interactions with the micelle (see below).

### Evidence for detergent-like action of A *β*

At SDS concentrations below the CMC, not membrane interactions, but individual detergent molecules, must be responsible for the solubilization of A *β*, i.e., the inhibition of aggregates. Put differently, detergent molecules must be incorporated into the A *β*-aggregates under these conditions, suggesting a detergent-like behavior of A *β* and the ability of A *β* or its aggregates to bind detergent molecules. Presumably, the A *β* peptide acts as a kind of detergent. Support comes from NMR results, which suggest a co-aggregate of detergent and A *β* with a hydrodynamic radius of 6 nm. A mixture of random-coil and *β*-sheet is found and involvement of the A *β*-N-terminus is concluded [23], similar to the present study.

## Conclusion

Overall, the present EPR investigation suggests that even at low concentrations SDS can inhibit A *β* aggregation by promoting A *β*-SDS complexes. Since, according to other techniques, this change is accompanied by a loss in *β*-sheet signature and an increase in *α*-helix character [21], we propose that this is the first step towards the micelle-bound state of A *β*, in which the monomeric peptide has an *α*-helical structure.

Apparently, aggregates bind detergent, which we extrapolate to suggest that there are forms of A *β* that can be membrane active, and this suggests that they could also bind lipid molecules, a hypothesis that is supported by the finding of a cholesterol-binding site of the A *β*-precursor, the APP protein [64, 65]. Whether such aggregates play a role in neuronal toxicity is one of the many open questions in A *β* research.

In conclusion, we have shown that previously inaccessible detail of the low-SDS form of A *β* can be obtained by spin-label EPR. A careful study of two labeling positions in A *β* and the sensitivity of this EPR approach to local mobility reveal a change in the aggregate state. From a particle, in which the N-terminus of A *β* participates in the solubilization and is located at the particle–water interface, the aggregate changes to an SDS-solubilized state that is a precursor to the one A *β*/micelle state above the CMC of SDS. Most striking is the observation that the N-terminus is active in the low D/P regime, where we suspect tight detergent–aggregate interaction. If lipid binding proceeds similarly, it may be that membrane interaction in a disease context involves the N-terminus, suggesting this region as a target to reduce membrane damage by A *β*. We stress that the submicellar SDS regime, in which A *β* is NMR-silent, mimics a situation that is relevant for the cellular action of A *β*. [21], namely a high (local) concentration of A *β* close to a membrane. With respect to the disease, these may well be sites where A *β* aggregation is initiated and our results suggest that aggregates formed at such sites could be membrane active, enabling us to speculate that membrane damage may result from such initial aggregates.

We demonstrate how from the local mobility parameters, not only the behavior of the N-terminal or the central positions of A *β* can be discriminated but also global properties of the A *β*-aggregation state are obtained, revealing the unique potential of EPR in studying the A *β* aggregation in situ.
